# Anatomical relationship between maxillary posterior teeth and the maxillary sinus in an Indonesian population: a CT scan study

**DOI:** 10.1186/s12903-024-04783-9

**Published:** 2024-08-29

**Authors:** Erli Sarilita, Rizky Maulana Muhammad, Harry Galuh Nugraha, Nani Murniati, Harmas Yazid Yusuf, Yohei Takeshita, Junichi Asaumi

**Affiliations:** 1 Department of Oral Biology, Faculty of Dentistry, Universitas Padjadjaran, West Java, Indonesia; 2 Dentistry Study Program, Faculty of Dentistry, Universitas Padjadjaran, West Java, Indonesia; 3 Department of Radiology, Faculty of Medicine, Universitas Padjadjaran, West Java, Indonesia; 4 Department of Oral Maxillofacial Surgery, Faculty of Dentistry, Universitas Padjadjaran, West Java, Indonesia; 5 Department of Oral and Maxillofacial Radiology, Dentistry and Pharmaceutical Sciences, Okayama University Graduate School of Medicine, Okayama, Japan

**Keywords:** Maxillary posterior teeth, Maxillary sinus, CT-Scan, Indonesia population

## Abstract

**Background:**

The anatomical proximity between the root apex of the maxillary posterior teeth and the maxillary sinus can lead to complications, including odontogenic maxillary sinusitis. While several studies have investigated similar variables in different populations, there is limited research on the Indonesian population. This study aimed to describe the anatomical position of the maxillary posterior teeth in relation to the floor of the maxillary sinus using CT scans.

**Methods:**

A total of 122 patients (432 maxillary premolars and 1,282 maxillary molars) underwent CT scans to evaluate 1,711 roots. The vertical relationship between the root apex of the maxillary posterior teeth and the maxillary sinus was classified into three types: IS (inside sinus), CO (sinus contact), and OS (outside sinus).

**Results:**

The IS type was predominantly found in the palatal roots of the first molars, accounting for 20% of the total roots in this type. The CO type was most frequently observed in the mesiobuccal roots of the second molars, representing 18% of the total roots in this type. The OS type was most commonly found in the first premolar, comprising 20% of the total roots in this type.

**Conclusions:**

The palatal roots of the first molars exhibited the highest frequency of proximity to the maxillary sinus. CT scans can effectively assess the relationship between the root apex of the posterior teeth and the maxillary sinus.

**Clinical Relevance:**

Clinicians should consider this information comprehensively when planning treatments for maxillary molars.

## Background

In the facial area, the maxillary sinus, the largest sinus, is closely related to dentistry. Anatomically, it is in close proximity to the apex of the maxillary posterior teeth [1–3]. During growth, the maxillary sinus can extend between the apex of the maxillary posterior teeth and a layer of cortical bone [4]. This close anatomical relationship between the maxillary posterior teeth and the maxillary sinus can potentially lead to odontogenic maxillary sinusitis. The causes of odontogenic maxillary sinusitis can include periodontal disorders, periapical abnormalities, or iatrogenic factors [5]. Odontogenic infections alone account for 10–12% of total cases of maxillary sinusitis [6]. If left untreated, it can spread to orbital and cranial structures [6].

Iatrogenic cases have shown the highest prevalence of odontogenic maxillary sinusitis [5, 7]. A study by Arias-Irimia et al. in 2010 found that 65.79% of the total 770 cases were iatrogenic [5]. Complications related to root canal treatment, such as over-instrumentation and intrusion of foreign bodies into the sinus cavity, can stimulate the occurrence of maxillary sinusitis [8]. Additionally, complications from extraction procedures, such as intrusion of root remnants into the sinus or oroantral communication, are also related to the proximity between the root canal of maxillary posterior teeth and the maxillary sinus [7]. A study by Lee et al. in 2010 reported that 8 out of 27 patients with odontogenic maxillary sinusitis were associated with post-extraction complications (29.6%) [9]. Therefore, understanding the proximity of these two structures is crucial for clinicians to minimize complications after root canal treatment or extraction procedures.

Radiographic techniques that can be employed include conventional or 3D radiographic techniques. However, conventional radiographic techniques have limitations in image resolution, which can result in distortion and superimposition [10]. The use of 3D radiographic techniques, such as CT scans, can overcome these disadvantages by providing sagittal, axial, and coronal sections of the radiographic image [6]. In a study by Sharan et al. in 2006 involving 80 patients, it was found that 6 out of 10 visible roots of teeth intruding into the maxillary sinus on panoramic radiographs did not accurately represent the true condition when observed using CT scan radiographs [11].

Previous studies on the relationship between maxillary posterior teeth and maxillary sinuses have been conducted in various populations, including China, Japan, Turkey, India, Iran, and Korea [8, 12–16]. Research involving populations outside the Asian continent has also been conducted [17, 18]. Studies with similar variables using panoramic radiography, CT scans, and CBCTs have been performed on various populations [12]. However, based on a systematic search of journals, no studies with similar variables have been conducted on populations in Indonesia using CT scans. Therefore, the aim of this study is to describe the anatomical position between the roots of maxillary posterior teeth and the floor of the maxillary sinus using CT scans.

## Materials and methods

This descriptive study utilized secondary data from patients who visited the Radiology Clinic of Hasan Sadikin Hospital (RSHS) Bandung between January and June 2019. The sampling was conducted cross-sectionally, and the sample size was determined using purposive sampling technique. Out of the total population of 266 CT scan images, a total of 122 patients (61 males and 61 females) aged between 19 and 76, with an average age of 41 ± 12.83 years, met the inclusion and exclusion criteria. The sample selection criteria included a minimum age of 18 years and clear CT scans showing intact and pathology-free images of the root apex of the maxillary permanent posterior teeth, along with healthy maxillary sinuses characterized by radiopaque cortical bone. Unerupted teeth, periapical disease, periodontal disease, alveolar bone abnormalities, and maxillary fractures were excluded from this study. The sample consists of 1711 roots of maxillary posterior teeth, ranging from the first premolar (P1), second premolar (P2), first molar (M1), second molar (M2), to the third molar (M3).

This is a secondary data research study that utilized anonymous medical imaging data. No additional radiation exposure was involved for research purposes, as the data was obtained from the initial diagnostic procedures conducted as part of the treatment planning in which mostly for stroke management. Routine informed consent was collected in accordance with the standard operating protocol in the Radiology Clinic of Hasan Sadikin Hospital. Ethical exemption was provided by the Research Ethics Committee of Universitas Padjadjaran (403/UN6.KEP/EC/2021).

### Image interpretation

**Fig. 1:**
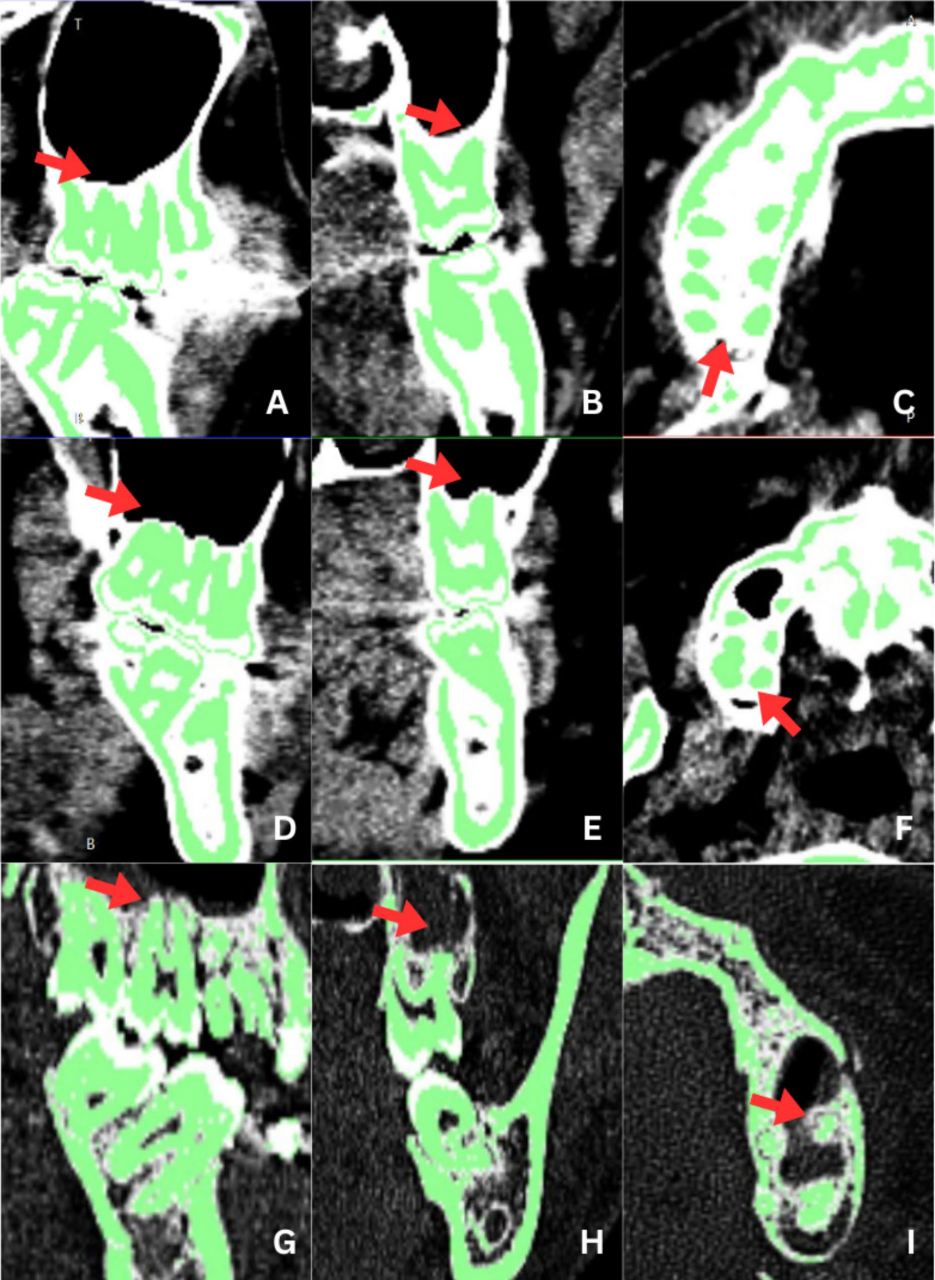
Classification of the relationship between the maxillary posterior teeth and the maxillary sinus floor (**A**, **B**, and **C** indicate the classification for the Outside Sinus (OS) type; **D**, **E**, and **F** represent the classification for the Contacting (CO) type. **G**, **H**, and **I** depict the classification for the Inside Sinus (IS) type)

The CT scan images were acquired using the SOMATOM Definition DS dual-source 128 CT scanner (Siemens, Erlangen, Germany) with a section thickness of 1.0–1.5 mm. The operating parameters were set at 110 kV and 10 mA, and the scans were irradiated for 18 s. The CT scan results were initially interpreted and subsequently re-evaluated by intra- and inter-observers on 15 randomly selected samples after a 2-week interval. The raters were two dental students under the supervision of three assistant professors. The position of the maxillary posterior roots in relation to the maxillary sinus was assessed in sagittal, axial, and coronal sections using Invesalius 3.10 software. Upon entering the digital CT scan data into the Invesalius software, the images were imported to visualize the relationship between the posterior tooth roots and the maxillary sinus floor in sagittal, axial, and coronal sections. To enhance image clarity and facilitate evaluation, dentin-type masking was performed with a value of 1400–2400 Hounsfield Units, and the brightness level of the CT scan was increased. For the purpose of this study, the relationship between the maxillary posterior tooth roots and the maxillary sinus floor was classified into three categories (Fig. 1). Type 1, or Inside Sinus (IS), indicated that the root tip intruded inside the maxillary sinus floor. Type 2, or Contacting (CO), denoted that the root tip was attached to the base layer of the maxillary sinus cortical bone. Type 3, or Outside Sinus (OS), indicated that the root tip was located slightly or below the maxillary sinus floor. Statistical processing was performed using SPSS 25.0 (IBM Corp., Armonk, NY, USA). To assess the reliability of the intra-observer and inter-observer, Cohen’s kappa test was utilized.

## Results

### Inter- and intra-observer reliability

The inter- and intra-observer reliability tests were conducted using Cohen’s kappa test statistics. The results obtained were above the threshold of 0.7, indicating moderate to perfect reliability (Table 1) [19].

**Table 1:** The results of the reliability test on the results of inter-observer and intra-observer observations

Data	Type of tooth root	Kappa Value
	P1	1.000
	P2	1.000
	M1MB (Mesiobuccal)	1.000
	M1DB (Distobuccal)	0.708
*Inter-observer*	M1P (Palatal)	0.835
	M2MB (Mesiobuccal)	0.724
	M2DB (Distobuccal)	0.721
	M2P (Palatal)	0.835
	M3	0.857
	P1	1.000
	P2	1.000
	M1MB (Mesiobuccal)	1.000
	M1DB (Distobuccal)	0.759
*Intra-observer*	M1P (Palatal)	0.868
	M2MB (Mesiobuccal)	0.707
	M2DB (Distobuccal)	0.777
	M2P (Palatal)	0.821
	M3	0.862

### Frequency of maxillary posterior roots in the maxillary sinus proximity classification

Table 2 presents the results of the vertical relationship between the maxillary posterior teeth and the maxillary sinus floor classification. For the P1 root, the majority (95%) belonged to the OS type, accounting for 209 out of 219 roots. The CO type and IS type accounted for 4% and 1%, respectively. In the case of the P2 root, the highest frequency (70%) was observed in the OS type, with 149 out of 213 roots. The CO type and IS type accounted for 28% and 2%, respectively. Moving on to the maxillary first molar (M1), the M1MB, M1DB, and M1P roots were predominantly classified as OS type, ranging from 52 to 61%. The CO type and IS type accounted for percentages ranging from 35 to 38% and 4–9%, respectively. Similarly, in the maxillary second molar (M2), the M2MB, M2DB, and M2P roots were mostly classified as OS type, with percentages ranging from 39 to 56%. The CO type and IS type accounted for percentages ranging from 37 to 55% and 6–7%, respectively. Lastly, for the maxillary third molar (M3), the OS type had the highest frequency, accounting for 51% (65 out of 127 teeth). The CO type and IS type accounted for percentages of 42% and 7%, respectively.

**Table 2:** Classification results of the vertical relationship between maxillary posterior teeth and maxillary sinus floor

Tooth	Type IS		Type CO		Type OS		Sum of Tooth Roots
	*n*	%	*n*	%	*n*	%	
P1	2	1	8	4	209	95	219
P2	5	2	59	28	149	70	213
M1MB	8	4	67	35	115	61	190
M1DB	9	5	71	37	110	58	190
M1P	18	9	73	38	99	52	190
M2MB	12	6	106	55	76	39	194
M2DB	12	6	91	47	91	47	194
M2P	14	7	71	37	109	56	194
M3	9	7	53	42	65	51	127
Total	89		599		1023		1711

### The highest frequency in the maxillary sinus proximity classification

In the IS type classification, the M1P root had the highest frequency, accounting for 18 out of 89 roots (20% of all roots classified as IS). In the CO type classification, the M2MB root had the highest frequency, with a total of 106 out of 599 roots (18%). On the other hand, in the OS type classification, the P1 root had the highest frequency, with a total of 209 out of 1023 roots (20%).

## Discussion

Sinus pneumatization is a natural process that increases the volume of the maxillary sinus [10, 20]. Maxillary sinus development occurs predominantly during the first and second decades of life, ceasing when the third molar root is fully formed between the ages of 18 and 25 years [11, 21]. However, pathological conditions such as tooth loss can lead to continued pneumatization and bone resorption [22]. As observed in this study, pneumatization tends to occur in an inferior and medial direction, increasing the proximity of the first and second molars to the maxillary sinus [10]. The anatomical structure of the maxillary sinus floor, which forms a curve with its lowest point near the roots of the first and second molars, further contributes to this proximity [13, 17, 23, 24]. The close relationship between these structures can give rise to complications, such as odontogenic maxillary sinusitis, which can occur due to procedural errors during root canal treatment or post-extraction oroantral communication [5].

In this study, the majority of maxillary posterior teeth were classified as OS type (outside sinus), accounting for 60% of the total 1711 roots. The CO type (sinus contact) accounted for 35%, while the IS type (inside sinus) accounted for 5% of the total roots studied. It is important to note that previous literature has indicated iatrogenic factors as the leading cause of odontogenic maxillary sinusitis [5, 6, 9]. This emphasizes the need for clinicians to have a comprehensive understanding of the anatomy between the maxillary sinus and maxillary posterior teeth to minimize potential complications [25].

Several studies conducted in different populations have shown variations in the frequency of each classification. For example, Pagin et al. in 2013 and Kilic et al. in 2010 reported different percentages for each classification, with IS type frequencies ranging from 14 to 21.60%, and CO type frequencies ranging from 14.3 to 21% of the total roots [17, 26]. These variations could be attributed to the use of cone-beam computed tomography (CBCT), which offer higher resolution and precision in assessing the relationship between the root apex of posterior teeth and the maxillary sinus. In the current study, M1P exhibited the highest frequency in the IS type classification (20% of the total 89 roots protruding into the sinuses).

Other variations could be attributed to ancestry group, although further research is still needed [27]. This study took samples of the East Asian ancestry group who naturally live in West Java [28]. It is worth noting that the sample in this study was taken from a provincial hospital in West Java, where the population is predominantly of East Asian ancestry, thus reflecting the diversity within this subgroup [29].

Similar findings have been observed in studies conducted in Turkey, China, and South Korea [12–14]. This study addresses a gap in the literature by exploring the anatomical relationship between maxillary posterior teeth and the maxillary sinus specifically in an Indonesian population. Previous studies have not examined this relationship within the unique subpopulation groups found in Indonesia, which differ from those studied in other populations.

Various studies in Asian populations using CBCT have reported similar results [12, 14, 22]. For instance, a study by Tian et al. in 2016, which included 848 Chinese patients, found that M1P had the highest frequency in the IS type classification (21% of 2121 total roots classified as IS), followed by M2MB (18% of 2121 total roots classified as IS) [27]. Another study by Ok et al. in 2014, involving 849 Turkish patients and 5166 roots, reported that M1P had the highest frequency in the IS type classification (34.2%) [14]. Similarly, Gu et al. in 2014, studying 1011 Chinese patients, and Jang et al. in 2017, studying 219 Korean patients, both using CBCT, found that the IS type was most commonly observed in the palatal roots of the first molars (24.8% and 15.93%, respectively) [12, 13].

However, studies by Pagin et al. in 2013 in Brazil and Oishi et al. in 2020 in Japan reported different findings. Pagin et al. found that M2MB had the highest frequency in the IS type classification, while Oishi et al. found that the IS type was most commonly observed in the mesiobuccal root of the second molar (70.5% of the total mesiobuccal roots of the second molars) [8, 17]. Estrela et al. in 2016, studying 202 Russian patients, and Shokri et al. in 2014, studying 110 Iranian patients, both using CBCT, reported that the IS type was most commonly observed in the maxillary second molars (5.3% and 40% of the total roots classified as IS, respectively) [16, 30]. These variations in the frequency of the IS type may be influenced by factors such as age, gender, sample characteristics, and imaging technologies used [27, 31].

In terms of the CO type classification, this study found that the mesiobuccal roots of the second molars had the highest frequency (18% of the total 599 roots classified as CO). These results are consistent with those of Tian et al. in 2016, who reported that the CO type was most commonly observed in M2MB and M2DB (43% and 39.91% of 1530 roots, respectively), and Pagin et al. in 2013, who found the highest frequency of the CO type in the second molars (41% of the total 130 roots in proximity to the maxillary sinus without elevation) [17, 27]. Previous studies have consistently highlighted the mesiobuccal root of the second molar as being in close proximity to the maxillary sinus floor [12, 13, 17, 26, 27, 30, 32].

The OS type classification was most commonly observed in the first premolars, which is consistent with the findings of Ok et al. in 2014, who studied 2680 premolars and 2486 molars in the Turkish population and found that 92.4% of the 1379 P1 teeth belonged to the OS type [14]. Similar conclusions have been reported in other studies as well [16, 26, 32]. A study on a Western Chinese population found that 64.8% of maxillary second premolars were not attached to the maxillary sinus. However, the average distance between the root tip and the sinus floor was only 2.47 mm, indicating a narrow space separating the two adjacent structures [33]. Therefore, any type of dental treatment involving the root of second premolars must be considered carefully.

It is important to consider comprehensive treatment approaches for maxillary posterior teeth, as periapical diseases such as apical periodontitis can lead to odontogenic maxillary sinusitis due to the spread of infection to adjacent anatomical structures [5, 6]. The roots of teeth that enter the sinus are only covered by a layer of cortical bone or may even be in direct contact with the sinus membrane. Therefore, pre-operative radiographs are crucial in assessing the relationship between the maxillary posterior roots and the maxillary sinus. While panoramic radiographs have been commonly used, studies have indicated potential biases, particularly in cases where roots appear to be intruding into the sinus laterally or medially [34]. CT scan images can help overcome distortion and superimposition present in panoramic radiographs, although they have limitations in terms of availability, radiation exposure, and cost [31]. CBCT is recommended for further research due to its higher resolution and ability to provide more accurate results in assessing the relationship between maxillary posterior roots and the maxillary sinus [8, 12, 14, 17, 27, 33, 35–38].

This study has some limitations, such as the assessment of distances and factors that may influence the relationship between roots and the maxillary sinus, including age, sex, and tooth region. In addition, the sample size was determined using a purposive sampling technique instead of a traditional sample size estimation method due to time constraints and the availability of CT imaging as the data source. This approach also helped avoid a prospective study, thereby eliminating unnecessary radiation exposure. Although CBCT is the preferred modality for this study, in the archipelagic context of Indonesia, CT scans are more widely available in terms of machinery and clinical expertise, making them a more practical choice for research and pre-treatment diagnostic procedures. Thus, this study offers a solution for examining the sinus floor using CT scans, which is suitable for clinical settings with limited resources. Further research using CBCT imaging technology and larger sample sizes within the Indonesian population are warranted to address these limitations.

## Conclusion

This study revealed that the majority of maxillary posterior tooth roots did not exhibit close proximity to the maxillary sinus floor. However, the palatal roots of the first molars were found to have the highest frequency of intrusion into the maxillary sinus. On the other hand, the mesiobuccal root of the second molar had the highest frequency of contact with the maxillary sinus. The root of the first premolar had the highest frequency of being outside the maxillary sinus, emphasizing the need for comprehensive consideration by dentists.

Sinusitis of odontogenic origin, stemming from inflammation of the maxillary teeth, is influenced by anatomical variants of the teeth roots. Therefore, the findings of this study are clinically relevant for dentistry, oral-maxillofacial surgery, and otolaryngology settings. Understanding these anatomical relationships can aid in diagnosing and treating sinusitis associated with dental conditions more effectively across these specialties.

## Data Availability

Availability of data and materialsAll the datasets used and analyzed during the current study are available from the corresponding author on reasonable request.

## Abbreviations

CBCT: Cone Beam Computed Tomography
CO: Contact Sinus
CT: Computed Tomography
IS: Inside Sinus
kV: Kilovolt
M1 P: Palatal root of upper first molar
M1 DB: Distobuccal root of upper first molar
M1 MB: Mesiobuccal root of upper first molar
M2 DB: Distobuccal root of upper second molar
M2 MB: Mesiobuccal root of upper second molar
M2 P: Palatal root of upper second molar
M3: Upper third molar
mA: Miliampere
mm: Milimeter
OS: Outside Sinus
P1: Upper first premolar
P2: Upper second premolar
SPSS: Statistical Package for the Social Sciences
